# Outbreak of Severe Acute Respiratory Syndrome Coronavirus 2 in a Rural Community Hospital during Omicron Predominance

**DOI:** 10.3390/microorganisms12040686

**Published:** 2024-03-28

**Authors:** Amar Krishna, Julie Tutt, Mehr Grewal, Sheila Bragdon, Suzanne Moreshead

**Affiliations:** 1 Northern Light AR Gould Hospital, Presque Isle, ME 04769, USA; jtutt@northernlight.org (J.T.); mehrgrewal28@gmail.com (M.G.); sbragdon@northernlight.org (S.B.); 2 Northern Light Health, Brewer, ME 04412, USA

**Keywords:** Omicron, severe acute respiratory syndrome coronavirus 2, coronavirus disease 2019, hospital outbreak, healthcare-associated infection, ultraviolet light disinfection

## Abstract

Healthcare-associated infections due to severe acute respiratory syndrome coronavirus 2 (SARS-CoV-2) has increased since the discovery of the Omicron variant. We describe a SARS-CoV-2 outbreak in the medicine–surgery unit of a rural community hospital at the time of high community transmission of Omicron variant in our county. The outbreak occurred in the medicine–surgery unit of an 89-bed rural community hospital in northern Maine. The characteristics of the patients and healthcare workers (HCWs) affected by the outbreak are described. Patient and HCW data collected as part of the outbreak investigation were used in this report. The outbreak control measures implemented are also described. A total of 24 people tested positive for SARS-CoV-2 including 11 patients and 13 HCWs. A total of 12 of the 24 (50%) persons were symptomatic, and rhinorrhea was the most common symptom noted (8/12, 67%). None of the symptomatic persons had gastrointestinal symptoms or symptoms of a loss of sense of smell or taste. All HCWs were vaccinated and 8 of the 11 patients were vaccinated. Outbreak control measures in the affected unit included implementation of full PPE (N95 respirators, eye protection, gowns and gloves) during all patient care, serial testing of employees and patients in the affected unit, cohorting positive patients, closing visitation and thorough environmental cleaning including use of ultraviolet (UV) light disinfection. This outbreak exemplifies the high transmissibility of the Omicron variant of SARS-CoV-2. The outbreak occurred despite a well-established infection control program. We noted that serial testing, use of N95 respirators during all patient care and UV disinfection were some of the measures that could be successful in outbreak control.

## 1. Introduction

Since its first emergence in China in December 2019, the severe acute respiratory syndrome coronavirus 2 (SARS-CoV-2) causing the coronavirus disease 2019 (COVID-19) has evolved into a global pandemic with over 774 million cases and 7 million deaths globally [1]. The virus has evolved and mutated continuously to produce variant strains that are different from the original SARS-CoV-2 virus. Most of these changes have little or no impact on the virus’s properties, but some of these changes can result in strains with increased transmissibility, increased virulence or show reduced effectiveness to current vaccines or therapeutics. These latter strains are designated as variants of concern (VOCs) to highlight their public health significance. The Omicron variant of SARS-CoV-2 was first reported to the World Health Organization (WHO) from South Africa in November 2021 [2]. This highly transmissible variant was designated as a VOC by the WHO on 26 November 2021 [2]. Since its first identification in South Africa, Omicron and its sub lineages have been detected in most countries leading to the exponential rise in SARS-CoV-2 infections in late December 2021 and early 2022.

In addition to infections acquired in the community, SARS-CoV-2 has resulted in a great number of healthcare-associated infections and outbreaks [3]. It is estimated that 10–20% of hospital diagnosed cases of COVID-19 are acquired in the hospital (healthcare-associated infections) [4,5,6]. By using epidemiological and sequencing data, a study in UK reported that majority of the SARS-CoV-2 infections occurring >7 days following hospital admission are nosocomially acquired [6]. The number of healthcare-associated SARS-CoV-2 infections has increased since the discovery of the Omicron variant [7]. This variant is about three times more transmissible than the Delta variant leading to the latter’s replacement as the dominant variant globally [8]. Increased transmissibility of the Omicron variant has been attributed to its immune evasion ability. In addition, changes in cell entry and cellular tropism might also contribute to increased transmissibility [8]. Vaccination and previous infection provide less protection against the Omicron variant compared to other variants [8].

Since the beginning of the pandemic, hospitals have adopted several administrative and engineering controls in addition to the use of personal protective equipment to prevent healthcare-associated SARS-CoV-2 infections. Surveillance testing for SARS-CoV-2 is an administrative control measure that has been used to detect asymptomatic and presymptomatic SARS-CoV-2 infections. Most commonly, screening is performed at the time of admission [9]. Some hospitals continue to screen patients during their hospital stay to detect infections that were incubating at the time of admission and, therefore, missed by initial admission screening or to detect infections acquired during the hospital stay [9].

SARS-CoV-2 outbreaks and clusters tend to occur in non-COVID-19 wards from patients and HCWs with unsuspected infections [10,11,12]. To counteract this, universal medical mask use has been adopted for respiratory protection and source control when caring for patients without suspected or confirmed COVID-19 [9]. With growing evidence of the airborne transmission of SARS-CoV-2 and outbreaks occurring despite universal medical mask use, some hospitals have adopted N-95 respirators for non- COVID-19 care especially during periods of high community transmission [9]. N-95 respirators (along with eye protection, gloves, and gown use) are also recommended for patients with COVID-19 or those undergoing aerosol generating procedures.

We share our experience on a SARS-CoV-2 outbreak in the medicine–surgery unit mostly housing non-COVID-19 patients at the time of Omicron predominance. The characteristics of the persons affected during the outbreak as well as the outbreak control measures implemented are described. We further evaluated factors that led to outbreak initiation and propagation.

## 2. Methods

The outbreak occurred in the medicine–surgery unit of an 89-bed rural community hospital in northern Maine. The medicine–surgery unit houses 27 beds consisting of 11 private rooms (including 2 airborne isolation rooms) and 8 semiprivate rooms. Each semiprivate room has two beds with separate privacy curtains for each bed.

The outbreak period was defined as the date when the first outbreak case tested positive for SARS-CoV-2 until no new cases were identified in the unit for 14 consecutive days since the date of the last positive test. An outbreak case was defined as any patient or healthcare worker (HCW) who tested positive for SARS-CoV-2 following exposure in the medicine–surgery unit during the outbreak period. SARS-CoV-2 testing of patients and HCWs were completed with different PCR tests, and nasal specimens were used for both PCR tests. Patient testing was performed using the cobas liat SARS-CoV-2 & Influenza A/B multiplex real-time RT-PCR (Roche Molecular Systems, Inc., Pleasanton, CA, USA) according to manufacturer instructions [13]. The assay is reported as positive for SARS-CoV-2 if it detects either or both of the two targets (open reading frame 1ab [ORF 1ab] non-structural region and nucleocapsid protein gene) of the SARS-CoV-2 genome [13]. HCW testing was performed using the Panther Fusion SARS-Cov-2 assay (Hologic Inc., San Diego, CA, USA). This assay targets two unique regions of the ORF 1ab section of the SARS-CoV-2 viral genome [14]. Patient and HCW data collected as part of the outbreak investigation were used in this report. This study was deemed quality improvement and exempted from full institutional board review.

When the outbreak occurred, the hospital had an established infection prevention program to prevent nosocomial SARS-CoV-2 transmission. The program included PCR testing for SARS-CoV-2 at the time of admission, universal surgical mask use (by HCWs, patients and visitors), the use of eye protection during patient care, mandatory employee vaccination requirement, employee and visitor symptom screening, daily symptom screening of hospitalized patients, visitor restriction (≤2 per day), free on-demand testing of symptomatic and exposed HCWs, contact tracing and exposure risk assessment, and an aerosol-generating procedure (AGP) policy.

## 3. Results

**Figure 1:**
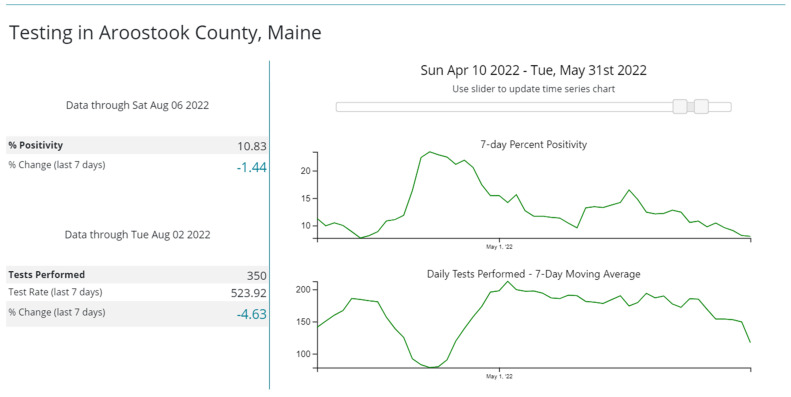
Percentage positivity of SARS-CoV-2 tests in Aroostook County, Maine, during April–May 2022. The top graph shows that in late April, the test positivity was >15% in the county. Downloaded from the Centers for Disease Control and Prevention COVID-19 Data Tracker.

The outbreak was suspected when two patients and two staff in the medicine–surgery unit tested positive for SARS-CoV-2 in late April 2022. At that time, the BA.2.12.1 subvariant of Omicron accounted for 96–100% of sequenced isolates in our county [15]. Community SARS-CoV-2 transmission in the county at that time was high with >15% test positivity rate (Figure 1).

**Figure 2:**
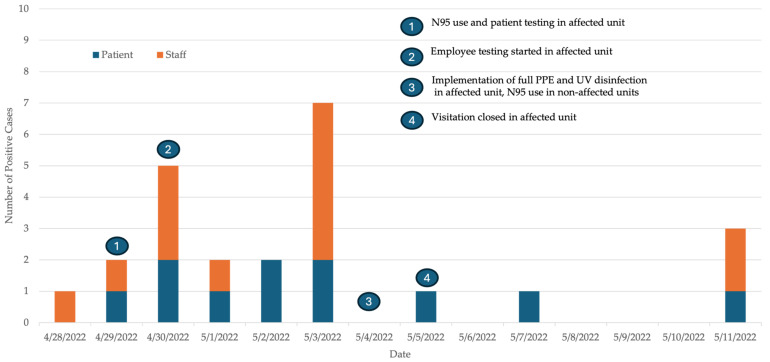
Epidemic curve of SARS-CoV-2-positive patients and staff along with the date of implementation of the various control measures during the SARS-CoV-2 outbreak in the medicine–surgery unit.

**Figure 3:**
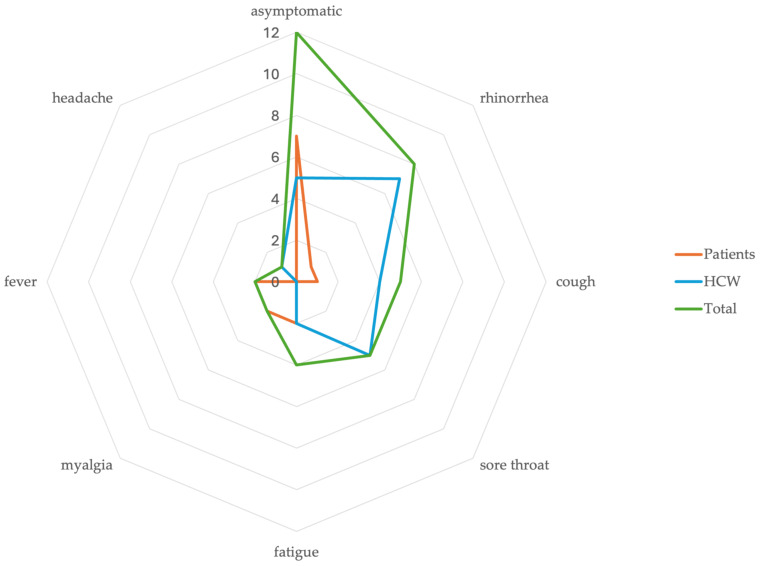
Graph indicating the number of patients and HCWs with various symptoms of SARS-CoV2 infection.

A total of 930 tests were performed during the outbreak period including 590 patient tests and 340 employee tests. Among the 85 patients and 53 HCWs tested during the outbreak, a total 24 people tested positive including 11/85 (13%) patients and 13/53 (24%) HCWs (Figure 2). A total of 12 of the 24 (50%) people were symptomatic including 4/11 (36%) patients and 8/13 (61%) HCWs. In symptomatic persons, the most common symptom was rhinorrhea 8/12 (67%) followed by cough 5/12 (42%) and sore throat 5/12 (42%) (Figure 3). None of the symptomatic persons reported the loss of sense of smell or taste or gastrointestinal symptoms. All HCWs (100%) and 8/11 (73%) patients were vaccinated with either the mRNA COVID-19 vaccines (all received at least two doses) or the single dose J&J/Janssen viral vector vaccine (Janssen Biotech, Inc., Horsham, PA, USA). Among vaccinated persons, most received (18/21, 86%) the mRNA COVID-19 vaccines and the rest (3/21, 14%) the J&J/Janssen viral vector COVID-19 vaccine.

The index case was likely a HCW who worked in the unit and had symptoms for about 1 week. The HCW had two antigen tests performed during this period, both of which were negative. The HCW later tested positive on a home antigen test. An alternate or concurrent index case could have been the patient who was hospitalized for 4 days in late April after testing negative on admission. This patient was discharged and readmitted a day later at which time admission testing was positive. However, without viral genome sequencing, these suspicions are merely speculative. Early in the outbreak, a tracheostomy patient who required frequent suctioning following COVID-19 diagnosis infected three HCWs, and infected HCWs failed to use appropriate personal protective equipment (PPE) during suctioning.

## 4. Outbreak Control Measures

Outbreak control measures in the affected unit included twice-weekly PCR testing of uninfected HCWs and patients, closing visitation, cohorting positive patients, quarantining exposed patients and implementation of full PPE (N95 respirators, eye protection, gowns and gloves) during all patient care. HCWs in the affected unit were allowed to work while awaiting test results if they were asymptomatic. Direct patient facing activities in other hospital areas required the use of N95 respirators and eye protection without the need for gowns and gloves. Thorough environmental cleaning was emphasized following patient discharge. In addition, ultraviolet (UV) light disinfection was performed after patient discharge to reduce viral load in the air and on environmental surfaces. We also used UV light disinfection of the breakroom and the family room located in the unit. The time of implementation of the various control measures during the outbreak is presented in Figure 2. With these measures, the outbreak was officially closed on 25 May, 14 days after the last positive case.

## 5. Discussion

This hospital outbreak emphasizes the high transmissibility of the Omicron variant even in a vaccinated population. The outbreak occurred despite a well-established infection control program.

About 50% of the infected persons were asymptomatic, which is similar to previous reports of infections with the Omicron variant [16,17,18]. We noted rhinorrhea, cough and sore throat as the predominant symptoms. Previous studies note a predominance of cold-like symptoms with Omicron infections compared to previous variants [19,20]. Symptoms of a loss of sense of smell or taste and gastrointestinal symptoms have been features of SARS-CoV-2 infections especially during the Delta and pre-Delta periods [21]. None of the symptomatic persons in this outbreak had a loss of sense of smell or taste and none had gastrointestinal symptoms. This is not surprising, as these symptoms might be less common with Omicron infections when compared to Delta and pre-Delta variant infections, as noted in a multi-institutional prospective cohort study [21]. The symptom profile of our outbreak population along with the isolation of the Omicron variant in the majority of the sequenced isolates in the county during the outbreak period strongly indicate that this variant was responsible.

The predominant use of surgical masks by our HCWs during the care of non-COVID-19 patients was likely insufficient during periods of high community transmission when a greater number of patients and HCWs can have asymptomatic and presymptomatic SARS-CoV-2 infections [22,23]. Evidence indicates that SARS-CoV-2 is mainly transmitted by the airborne route and, therefore, respirators provide better protection [24]. Even though it is not considered high-risk exposure if both patients and HCWs are wearing facemasks, we noted inconsistent mask use by patients [25]. Considering all these factors, the institution of respirators becomes necessary regardless of a patient’s infection status during periods of high community transmission as recommended by the Centers for Disease Control and Prevention (CDC). Due to the possibility of patients incubating the virus at time of admission and, therefore, testing negative on admission testing, repeat testing 48–72 h following admission should be considered. Following our outbreak, we started testing patients who tested negative on admission with a repeat test on day three to identify such cases.

When an outbreak is suspected, the serial testing of patients and HCWs is a crucial control measure, as it helps identify additional positives who may or may not be symptomatic [7]. Due to limited test availability, we performed twice-weekly testing to identify new infections, probably prolonging the outbreak. Others have shown that more frequent testing such as daily testing might lead to faster outbreak control by identifying positive cases earlier [26].

AGPs are a risk factor for the transmission of SARS-CoV-2 in the healthcare setting [27]. Open suctioning of airways is classified as an AGP by the CDC and this procedure was included in our AGP list requiring use of N95 respirators (along with eye protection, gloves, and gown use) and appropriate air exchange. Despite this, we noticed that infected HCWs reported not using appropriate PPE during tracheal suctioning. It appears that either the HCWs were unaware of this recommendation or were complacent as they perceived a low risk of SARS-CoV-2 infection in the tracheostomy patient who was hospitalized for several months and did not receive any visitors. This underscores the importance of frequent HCW education on appropriate PPE use during AGPs and monitoring adherence to these recommendations.

Previous in vitro studies have demonstrated the efficacy of UV light disinfection against SARS-CoV-2 on environmental surfaces and respirators [28,29,30]. UV-C disinfection has also been used in the prevention of other hospital-acquired infections such as *Clostridioides difficile* and Methicillin resistant *Staphylococcus aureus* [31]. To our knowledge, this is the first study to use UV disinfection of SARS-CoV-2 in the context of an outbreak.

Limitations of our study include relying solely on epidemiological investigation without whole genome sequencing, which might have resulted in wrongly attributing cases acquired in the community to the outbreak [32]. However, several of these cases occurred close together with clear exposure to persons in the outbreak making this less likely. Without WGS, it is unclear if this was a point source outbreak (single index case) or if multiple instances of viral introduction were responsible [32]. We also adopted several outbreak control measures concurrently and, therefore, were unable to discern the relative efficacy of each intervention.

## 6. Conclusions

Our experience is unique in that the outbreak occurred in a rural community hospital where limited resources and staff shortages can pose significant challenges. We noted that the lack of awareness about AGPs and limited testing capacity likely contributed to outbreak initiation and propagation in our rural community hospital. Future studies should explore whether similar factors are responsible for SARS-CoV-2 outbreaks in smaller community hospitals. Based on our experience, N95 respirator use especially during periods of increased community transmission, serial testing of patients and HCWs, appropriate PPE use during AGPs even when SARS-CoV-2 infection is not suspected, and post admission testing and monitoring of all inpatients are some of the measures that could prevent or limit future outbreaks. Future studies should explore the utility of UV light disinfection in the prevention of healthcare-associated SARS-CoV-2 infections.

## Data Availability

The data presented in this study is available on request from the corresponding author. The data are not publicly available due to privacy restrictions.
